# London Hospital

**Published:** 1830-04

**Authors:** 


### LONDON HOSPITAL.

### *Ossification of the left Auriculo-ventricular Valves, after repeated Attacks of Rheumatic Gout.*

John Jones, æt. twenty-two, of a spare habit and sanguineous temperament, with a pale countenance and projecting sternum, commonly denominated chicken-breasted, was admitted into the hospital on the 17th October, under the care of Dr. Billing. He states that he has at different periods been attacked with rheumatic gout, both in the upper and lower extremities; and that about fifteen months ago he was entirely deprived of the use of his limbs for a fortnight. He suffered much about five months ago with dyspnœa, attended by a slight cough, and with palpitations at the heart, which have increased much within the last three months, and are always aggravated by the least motion. On applying the ear to the parietes of the chest, over the left side of the heart, the bellows sound is distinctly heard, and an impulse is communicated to the head of the observer. Pulse at the wrist ninety-six, and small; appetite good; much thirst; night sweats. Bowels relieved four or five times daily; urine free and clear.—V.S. ad ℥xvi. Vesic. reg. cordis.

He bore the bleeding well. Breathing is easier; palpitations not so frequent, and the bellows sound is less noisy. Pulse eighty-four.—Infus. Gent. comp. ter die.—Cal. c. Rheo. ℈i. alt. aur.

20th.—Sleeps well, and continues improving.—Contin. med.

24th.—Cough has increased since the 20th.—Infric. Ung. Ant. Tart. reg. cordis. Cont. med.

27th.—The ointment has produced an eruption. Cough very troublesome, and prevents his sleeping; countenance very pale. The bellows sound continues, and a double pulsation is now felt at the wrist.—Pule. Opii gr. i. ter die. Ol. Ricini omni mane si opus sit. Rept. Ung. Ant. Tart. Omitt. alia med.

31st.—Orthopnœa, chiefly at night; cough more severe.

November 3d.—Is now very much distressed by the great difficulty of breathing.—Setaceum reg. cordis.

7th.—Incessant vomiting came on yesterday, which was in some degree relieved by a dose of the Tinctura Opii.

8th.—He died last night at twelve o'clock.

*Post-mortem examination.* Thorax: The pleura costalis was found adhering to the pleura pulmonalis, and in some places the adhesions were exceedingly firm. The pericardium adhered throughout to the surface of the heart. The right auricle was healthy; the right ventricle was a little hypertrophied, and the internal coat of the pulmonary artery was very red.

The left auricle was considerably hypertrophied; the left auriculo-ventricular valves were so much ossified as to leave a small fissure, into which the point only of the dissecting scalpel could be introduced. The left ventricle was slightly hypertrophied, and the ostium of the aorta, as also its thoracic portion, appeared of a less caliber than natural. The lungs were much congested.

Abdomen: A little fluid was found in the abdomen; the liver was enlarged, and gorged with blood. The other viscera were healthy.

Head was not examined.

---

### *Disease of the Heart after Rheumatism.*

Joseph Howard, æt. forty, a weaver, emaciated, countenance of a leaden hue, and expressing great anxiety, was admitted on the 4th of February, under the care of Dr. Billing. He reports that he had been the subject of rheumatism about fourteen months ago, and that his present illness came on about ten months ago, with severe pain in the left side of the chest, after lifting a heavy weight: he obtained relief by being repeatedly cupped, and by taking mercurial pills until ptyalism was produced.

He now complains of dyspnœa, which is much increased by motion; of pain, and a feeling of tension in the region of the heart, and of some tenderness on pressure; and of a great tendency to syncope after fits of coughing, which are very violent, and attended by a slight mucous expectoration. The ronchus sonorus gravis and mucosus are heard on both sides of the chest; the respiration is puerile on the right side, and indistinct on the left; percussion gives a duller sound on the left side than on the right; and the left side is found to give half an inch more than the right, on measuring from the spine of the vertebra to the xyphoid cartilage. The heart's pulsation is felt over a considerable extent of the anterior thoracic parietes, and the bellows sound is heard on applying the stethoscope over the situation of the left side of the heart. Decubitus always on the left side. Pulse 120, hard and irregular.—V.S. ad ℥xvi. statim. Mist.* Ant. Tart. ℥ss. om. horâ. Milk diet.

5th.—He feels a little better to-day. Had one very copious loose motion, and was sick in the night; nausea is produced by the medicine. Cough not so troublesome; respiration the same as yesterday, but with less ronchus; heart's action continues the same; pulse 120, hard, and weaker than yesterday.—Omittr. medicamenta. Tinct. Digit. ʒss. ter die, ex Infus. Gent. comp. Vesic. lateri dolente. Middle diet and rice pudding.

9th.—Less pain of side; cough very troublesome; dyspnœa is now increased to orthopnœa: lips somewhat livid; great faintness, and he feels very chilly; respiration laborious, and about sixty in a minute; action of the heart the same; pulse 120, stronger, and vibrating; bowels free; no appetite.—Hirudines xx. reg. cordis. Omittr. Tinct. Digit. Liq. Hydrarg. Oxym. ʒij. omni horâ. Liq. Opii Sed. gtt. xxx. h. s.

10th.—Experienced some benefit from the application of the leeches, but, in consequence of the pain still continuing, sixteen were again applied in the evening, which relieved him entirely. Has coughed only three times since yesterday evening. The respiration is now tranquil, and twenty in a minute; can now lie down in bed, and continues on the left side. The respiratory murmur is still puerile on the right side, and indistinct on the left, and without any ronchus; the bellows sound is now clearer; pulse 108 and soft; bowels free; tongue white.

11th.—Did not sleep well last night. Cough more troublesome and dyspnœa greater than yesterday; tongue coated; bowels once relieved this morning; pulse 120, very hard and vibrating; face

* Half an ounce of the mixture contains one fourth of a grain of Ant. Tart.

appears more bloated and livid. The medicine produces nausea, but not vomiting. He feels so weak as not to be able to sit up in bed.—Omittr. Liq. Hydrarg. Oxymur. Infric. cruribus Ungt. Hydrarg.

12th.—Is free from pain to-day; feels faint and low; pulse 120, weak, but jerking; respiration still hurried; the lips are less livid; skin temperate and soft; tongue white and more furred; anorexia; very little expectoration. Yesterday, when the breathing became much oppressed, and the lips very livid, twelve leeches were applied to the scrobiculus cordis, and he took Tinct. Digit. ʒi.; Tinct. Hyosciam. ʒij.; Tinct. Camph. comp. ʒij. ex aqua. Tinct. Digit. ʒss. ex Aqua Menth. ter die. Low diet; beef-tea, table beer.

13th.—Slept very comfortably last night, and continues much the same as yesterday. In the evening he had ℥iss. of port wine, on account of the constant nausea.

14th.—The digitalis having produced its depressing effect in a great degree, with constant nausea and anorexia, pulse eighty-eight, and respiration fifteen, it was left off. In consequence of the soreness of the gums and throat, let the Ung. Hydrarg. be discontinued.—Acid. Hydrocyan. ♏iij. ex Aqua Cinnam. ter die.

16th.—Yesterday the hydrocyanic acid produced sickness, which was relieved by three doses of the effervescent mixture, with ten minims of the Tinctura Opii in each. The first dose of the acid produced sickness to-day. He slept better last night, and feels easier to-day. Pulse 108, regular, and much softer; lips less livid; gums still sore; respiration about thirty; bowels free; tongue clean.—Acid. Hydrocyani ♏iij.; Quinæ Sulph. gr. i. ex Infus. Rosæ ter die.

19th.—Has been improving during the last two days. Pulse 108 and full; appetite better; bowels free.—Elaterii gr. ss. alt. auroris. Cont. cætera.

23d.—Feels rather stronger to-day than he did yesterday; decubitus still on the left side; there is considerable fulness in the epigastric region. Pulse 120, full. Each dose of the Elaterium produces eight or ten motions.—Hirudines viij. scrob. cordis alt. auroris. Omit. Elaterium.

26th.—Eyelids becoming anasarcous. Breathing rather freer; cough less; appetite better; tongue furred; thirst; bowels open; urine high-coloured and scanty; pulse 108, strong and throbbing. —V.S. ad ℥xij. Pil. Hydrarg. gr. v. ter die. Omit. cætera.

27th.—The blood extracted yesterday was both buffed and cupped. He slept rather better during the night, but is much the same as yesterday: less anasarca of the face; pulse 120 and softer; respiration is still puerile on the right side; urine increased in quantity. To-day, by means of the stethoscope, pectoriloquy has been discovered at the superior spinal fossa of the scapula.—Cont. Pil.*

* Medical Gazette.

